# Head-to-Head Comparison of ^68^Ga-PSMA-11 PET/CT and Multiparametric MRI for Pelvic Lymph Node Staging Prior to Radical Prostatectomy in Patients With Intermediate to High-Risk Prostate Cancer: A Meta-Analysis

**DOI:** 10.3389/fonc.2021.737989

**Published:** 2021-10-20

**Authors:** Xueju Wang, Qiang Wen, Haishan Zhang, Bin Ji

**Affiliations:** ^1^ Department of Pathology, China-Japan Union Hospital of Jilin University, Changchun, China; ^2^ Department of Nuclear Medicine, China-Japan Union Hospital of Jilin University, Changchun, China; ^3^ Department of Surgery, China-Japan Union Hospital of Jilin University, Changchun, China

**Keywords:** ^68^Ga-PSMA-11 PET/CT, multiparametric MRI, pelvic lymph node metastases, sensitivity, diagnostic accuracy

## Abstract

**Purpose:**

To compare the diagnostic performance of ^68^Ga-PSMA-11 PET/CT and mpMRI for pelvic lymph node staging prior to radical prostatectomy in prostate cancer (PCa) patients based on per patient data.

**Methods:**

PubMed and Embase databases were searched until October 2020 for eligible studies evaluating head-to-head comparison of ^68^Ga-PSMA-PET/CT and mpMRI for the detection of pelvic lymph node metastases (PLNMs) using pelvic lymph node dissection (PLND) as gold standard. The pooled sensitivity, specificity, and area under the summary receiver-operating characteristics curve (AUC) were determined for the two imaging modalities.

**Results:**

Nine studies with 640 patients were included. The pooled sensitivity, specificity, and AUC for ^68^Ga-PSMA-11 PET/CT vs. mpMRI were 0.71 (95% CI: 0.48–0.86) vs. 0.40 (95% CI: 0.16–0.71), 0.92 (95% CI: 0.88–0.95) vs. 0.92 (95% CI: 0.80–0.97), and 0.92 (95% CI: 0.88–0.95) vs. 0.82 (95% CI: 0.79–0.86), respectively. There was substantial heterogeneity for both imaging modalities, and meta-regression analysis revealed that the number of patients, prevalence of PLNMs, PSA level, reference standard, and risk classification might be the potential causes of heterogeneity.

**Conclusion:**

This meta-analysis of head-to-head comparison studies confirms that there is a trend toward a higher sensitivity and diagnostic accuracy of ^68^Ga-PSMA-11 PET/CT compared to mpMRI for the detection of PLNMs in PCa patients. Nevertheless, according to current guidelines, PLND still needs to be recommended in case of negative results from ^68^Ga-PSMA-11 PET/CT due to significant risk of malignancy.

## Introduction

Correct lymph node staging is crucial to identify prostate cancer (PCa) patients with poor prognosis who would benefit from additional therapies (1, 2). Pelvic lymph node dissection (PLND) represents the gold standard, but it is impeded by increased risk of complications such as lymphedema and venous thromboembolism as well as longer hospital stay (3, 4). Although cross-sectional abdominopelvic imaging has been recommended for patients with intermediate to high-risk PCa across guidelines, conventional imaging techniques only have modest diagnostic accuracy (4–7).

In recent years, positron emission tomography (PET) techniques with PSMA ligands have emerged as a promising tool for PCa detection, tumor staging, and treatment planning (8). Among them, ^68^Ga-PSMA-11 and ^18^F-DCFPyL have been consecutively approved by the FDA for patients with primary and recurrent PCa (9, 10). Nevertheless, although ^18^F-based tracers offer important advantages such as higher production capacity, longer physical half-life, and minimal radiotracer accumulation in the bladder (11–13); up until now, ^68^Ga-PSMA-11 is still worldwide the most commonly used and provides the absolute majority of evidence in the literature for PSMA imaging. Importantly, many accuracy studies and two previous meta-analyses have reported favorable diagnostic performance of ^68^Ga-PSMA-11 PET/CT for the detection of pelvic lymph node metastases (PLNMs) in intermediate to high-risk PCa (14–17).

Multiparametric MRI (mpMRI), which combines T2-weighted imaging (T2WI), diffusion weighted imaging (DWI), and dynamic contrast-enhanced (DCE) sequence, has been the leading imaging modality in the primary PCa detection and localization in the last decade. Several previous studies have compared it with ^68^Ga-PSMA-11 PET/CT for pelvic lymph node staging prior to radical prostatectomy. However, the results were variable and sometimes conflicting (18–32). Therefore, to clarify their relative effectiveness, in the present study, we sought to compare the diagnostic performance of these two imaging modalities by summarizing the most recent evidence in the literature. To reduce interstudy heterogeneity, only studies in which both modalities were performed in the same population were included.

## Material and Methods

This study was conducted according to the Preferred Reporting Items for Systematic Reviews and Meta-Analyses guidelines (33).

### Search Strategy

We comprehensively searched all available literature until October 2020 in the PubMed and Embase databases using an algorithm based on a combination of terms: (1) “Gallium Radioisotopes” (Mesh) OR Ga OR gallium; (2) “68Ga-PSMA” (Supplementary Concept) OR PSMA OR “prostate specific membrane antigen”; (3) “Positron Emission Tomography” (Mesh) OR PET OR “positron emission tomography”; (4) “Multiparametric Magnetic Resonance Imaging” (Mesh) OR mpMRI OR “Magnetic Resonance Imaging” (Mesh) OR “magnetic resonance imaging” OR MRI; (5) prostat*; (6) “Prostatic Neoplasms” (Mesh) OR pCa OR cancer* OR tumor* OR carcinoma; (7) “Lymph Nodes” (Mesh) OR “lymph node*” OR “lymph nodal” OR “locoregional.” The reference lists of identified publications were also hand-searched for potentially relevant studies.

### Inclusion and Exclusion Criteria

Studies were eligible for inclusion if all the following criteria applied: (a) the diagnostic performance of ^68^Ga-PSMA-11 PET/CT and mpMRI for pelvic lymph node staging prior to radical prostatectomy in PCa patients were clearly identified in the study or subset of the study; (b) the data were sufficient (i.e., patient number above 9) to construct a 2×2 contingency table; (c) the reference standard was histopathology confirmation from PLND, which should be clearly stated in the article. The exclusion criteria were (a) duplicated articles; (b) abstract, editorial comments, letters, case reports, review, or meta-analyses; and (c) clearly irrelevant titles and abstracts.

Using the aforementioned inclusion and exclusion criteria, two researchers independently screened titles and abstracts of the retrieved articles and then evaluated the full-text version of the remaining articles to determine their eligibility for inclusion. Disagreements between the researchers were resolved by consensus.

## Quality Assessment

Two researchers independently assessed the quality of the included studies based on the Quality Assessment of Diagnostic Accuracy Studies (QUADAS-2) tool. Each study was evaluated based on the following domains: patient selection, index test, reference standard, and flow and timing. These domains were then evaluated according to the risk of bias and were rated regarding applicability as “high,” “low,” or “unclear.” Disagreements between the researchers were resolved by consensus.

### Data Extraction

Two researchers independently conducted data extraction for all included articles. The extracted data included the first author, study characteristics (year, country, study design, prevalence of PLNMs, extracted lymph node number, and reference standard), patient characteristics (number of patients, age, PSA level, and D’Amico risk stratification), and technical aspects (field strength and MRI sequence for mpMRI; injection dose, uptake time, and image analysis for ^68^Ga-PSMA-11 PET/CT). For each study, the absolute numbers of true-positive, true-negative, false-positive, and false-negative data for mpMRI and ^68^Ga-PSMA-11 PET/CT were extracted on a per-patient basis. Disagreements between the researchers were resolved by consensus.

### Statistical Analysis

The pooled sensitivity and specificity for ^68^Ga-PSMA-11 PET/CT and mpMRI were presented as estimates with 95% confidence intervals (CIs) by using random-effect analysis. The summary receiver-operating characteristic (SROC) curves were constructed, and the area under the curve (AUC) was calculated.

Heterogeneity among pooled studies was assessed by use of Cochrane Q and *I* ^2^ statistics. Values of *I* ^2^ equal to 25, 50, and 75% were assumed to represent low, moderate, and high heterogeneity, respectively. In case of substantial heterogeneity, meta-regression analysis was performed to explore the potential source of heterogeneity and the covariates were (1) number of patients included (>40 vs. ≤40); (2) ethnicity (Asian vs. the rest); (3) prevalence of PLNMs (>20% vs. ≤20%); (4) extracted lymph node number (>10 vs. ≤10); (5) reference standard (PLND vs. extended PLND); (6) PSA (>10 vs. ≤10); (7) D’Amico risk stratification (high risk vs. intermediate and high risk); (8) PET image analysis (visual vs. quantitative); (9) field strength (1.5 T vs. 3.0 T); and (10) MRI sequence (T2WI, DWI, and DCE vs. DWI and DCE). Publication bias was assessed by Deeks’ funnel plot. All analyses were conducted with Stata 15.1 (Stata Corporation).

## Results

### Literature Search and Study Selection

**Figure 1:**
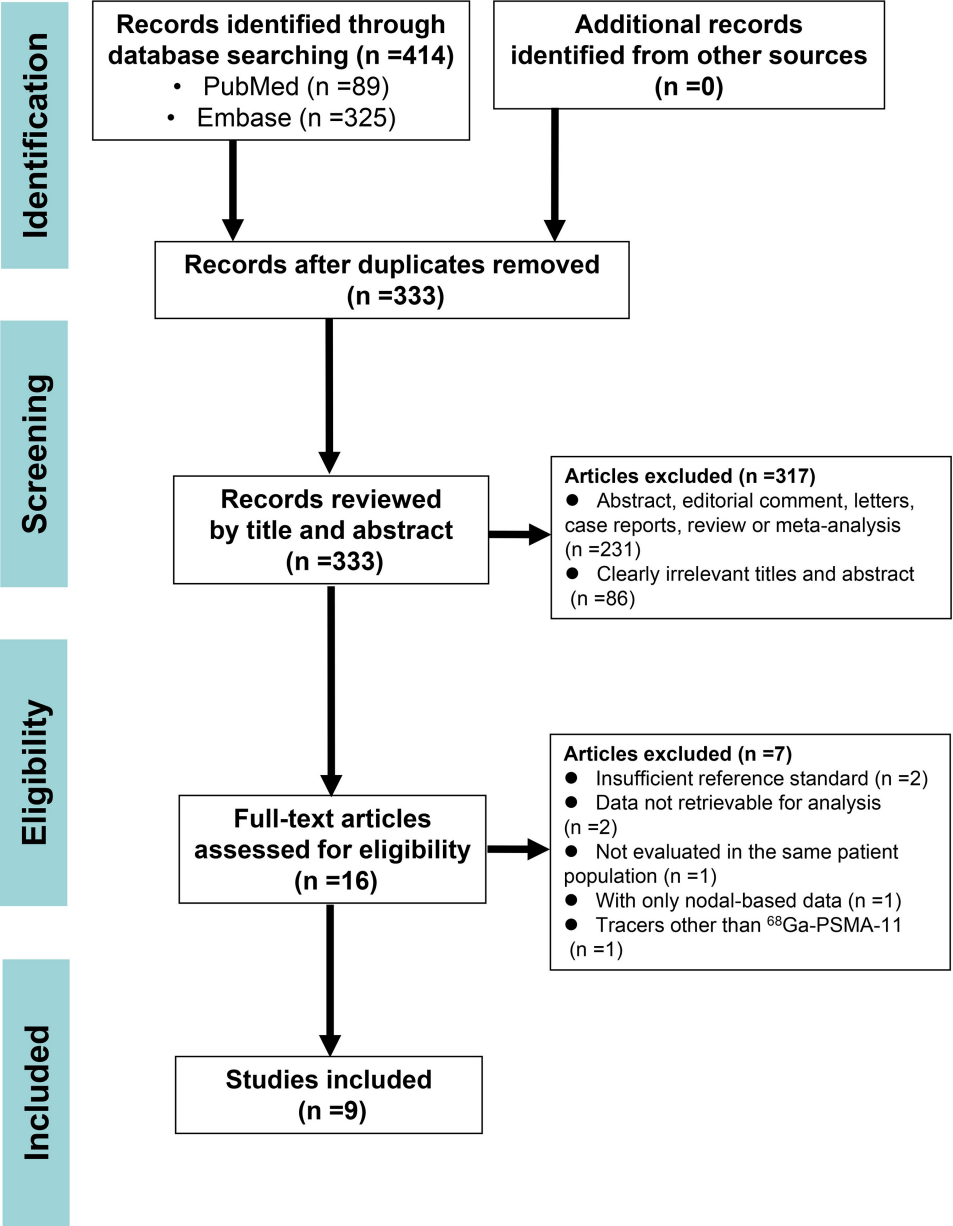
The PRISMA flow diagram of study selection.

The initial search retrieved 414 articles, and 398 were excluded upon review of titles and abstracts. The remaining 16 articles were carefully assessed by full text, and another seven were excluded for the following reasons: insufficient reference standard (n = 2); data not retrievable for analysis (n = 2); not evaluated in the same patient population (n = 1); with only nodal-based data (n = 1); and tracers other than ^68^Ga-PSMA-11 (n =1). Finally, nine articles including patient-based data on the head-to-head comparison of diagnostic performance of ^68^Ga-PSMA-11 PET/CT and mpMRI were eligible for further analysis. A PRISMA flow diagram of the study selection process is shown in **Figure 1**.

### Study Description and Quality Assessment

The study and patient characteristics of the nine articles comprising 640 patients are summarized in **Table 1**. The range of the prevalence of PLNMs for the included studies was 4% to 58.3%, and the median was 25%. The technical aspects of ^68^Ga-PSMA-11 PET/CT and mpMRI were presented in **Table 2**.

**Table 1:** Study and patient characteristics of the included studies.

Author	Year	Study characteristics					Patient characteristics			
		Country	Study design	Prevalence of PLNMs	No. of extracted lymph node	Reference standard	No. of patients	Age (median, range)	PSA (median, range)	D’Amico risk stratification
*Frumer et al. *(28)	2020	Israel	Retro	13.5%	Median 9 Range 6–14	PLND	89	67 (64–70)	8.5 (5–15)	Intermediate 40; high risk 49
*Franklin et al.* (32)	2020	Australia	Retro	24.5%	Median 16 Range 1–53	PLND	233	68 (48–81)	7.4 (1.5–72.0)	Low risk 2; intermediate 90; high risk 141
*Kulkarni et al. *(26)	2020	India	Retro	45.7%	NA	PLND	35	NA	NA	Intermediate and high risk
*Pallavi et al. *(31)	2020	India	Pro	24.1%	NA	NA	29	NA	NA	NA
*Van Leeuwen et al.* (24)	2019	Netherlands and Australia	Retro	36.4%	Median 16 Range 12–21	ePLND	140	NA	9.4	Intermediate 30; high risk 110
*Yilmaz et al.* (23)	2019	Turkey	Retro	20.0%	NA	rPLND	10	NA	NA	Low risk 3; intermediate 15; high risk 6
*Berger et al. *(21)	2018	Australia	Retro	4%	Median 12 Range 3–22	PLND	50	649 ± 5.6	10.6 ± 8.1	NA
*Gupta et al.* (20)	2017	India	Retro	58.3%	Median 20	ePLND	12	61 (46–76)	24.3 (8.7–200.6)	High risk 12
*Zhang et al. *(19)	2017	China	Retro	35.7%	Median 7 Range 2–15	PLND	42	69 (55–82)	37.25 (7.2–348.)	Intermediate 17; high risk 25

NA, not available.

**Table 2:** Technical aspects of ^68^Ga-PSMA-11 PET/CT and mpMRI scans.

Author	Year	mpMRI		^68^Ga-PSMA-PET/CT		
		Field strength	MRI sequence	Injection dose	Uptake time (min)	Image analysis
*Frumer et al.* (28)	2020	3.0 or 1.5 T	T2WI, DWI, DCE	3–5 mCi	50–60	Visual
*Franklin et al.* (32)	2020	3.0 T	T2WI, DWI, DCE	Mean, 200 MBq	45–60	Visual
*Kulkarni et al.* (26)	2020	3.0 T	T2WI, DWI, DCE	3–4.5 mCi	60	Visual
*Pallavi et al.* (31)	2020	3.0 T	T2WI, DWI	Mean, 1.76 MBq/kg	60	Visual
*Van Leeuwen et al.* (24)	2019	3.0 or 1.5 T	T2WI, DWI, DCE	2.0 MBq/kg or 100 MBq	60 or 45	NA
*Yilmaz et al.* (23)	2019	3.0 T	T2WI, DWI, DCE	Median, 175 MBq	60	Quantitative
*Berger et al.* (21)	2018	3.0 T	T2WI, DWI	NA	60	Quantitative
*Gupta et al.* (20)	2017	1.5 T	T2WI, DWI	2 MBq/kg	60	Visual
*Zhang et al.* (19)	2017	3.0 T	T2WI, DWI, DCE	Median 131.7 MBq	60	Visual

**Figure 2:**
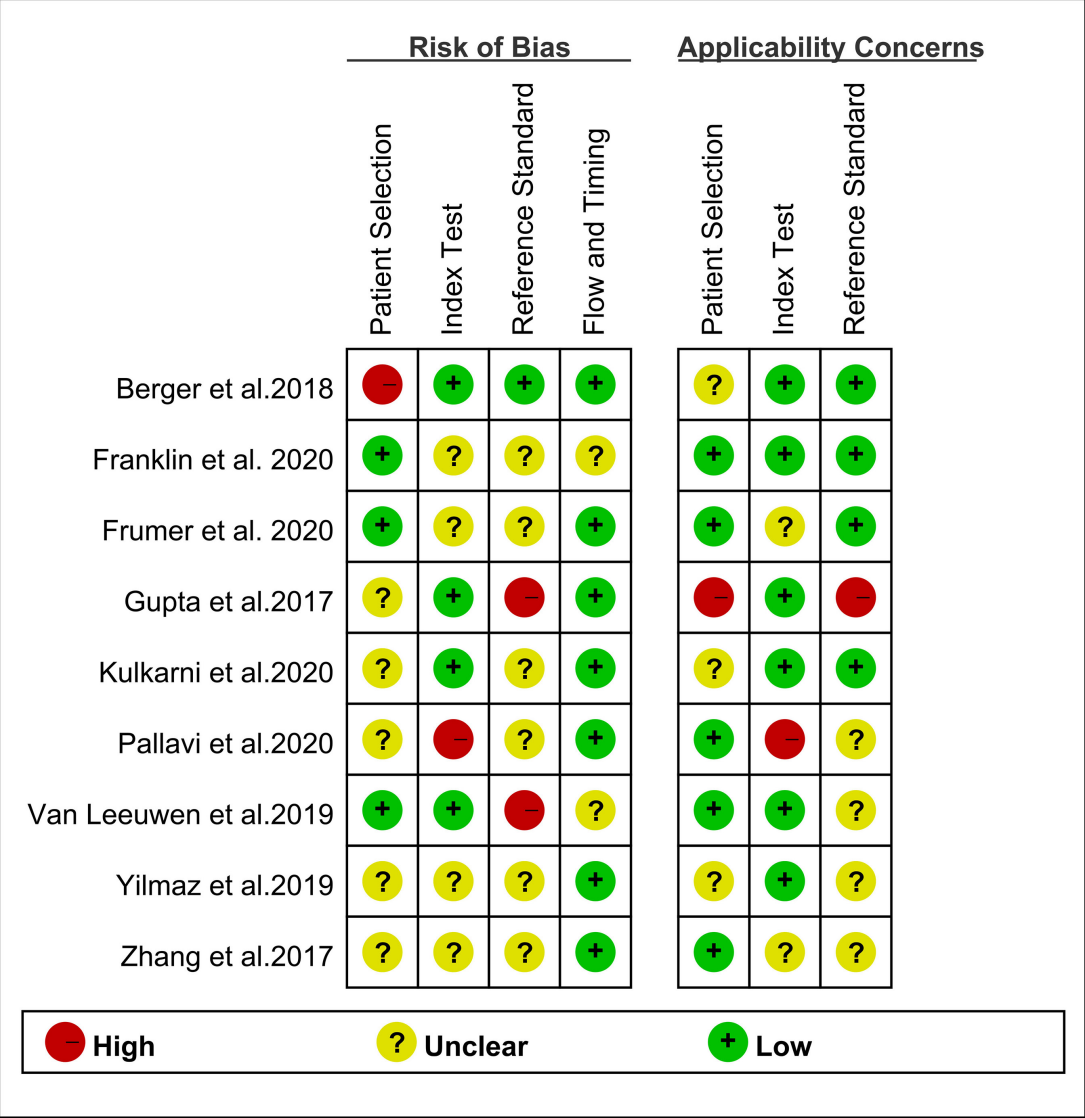
Summary risk of bias and applicability concerns of the included studies.

The results of summary risk of bias and applicability concerns of each study are shown in **Figure 2**. The quality of the included studies was considered satisfactory.

### Diagnostic Performance of ^68^Ga-PSMA-11 PET/CT for PLNMs

**Figure 3:**
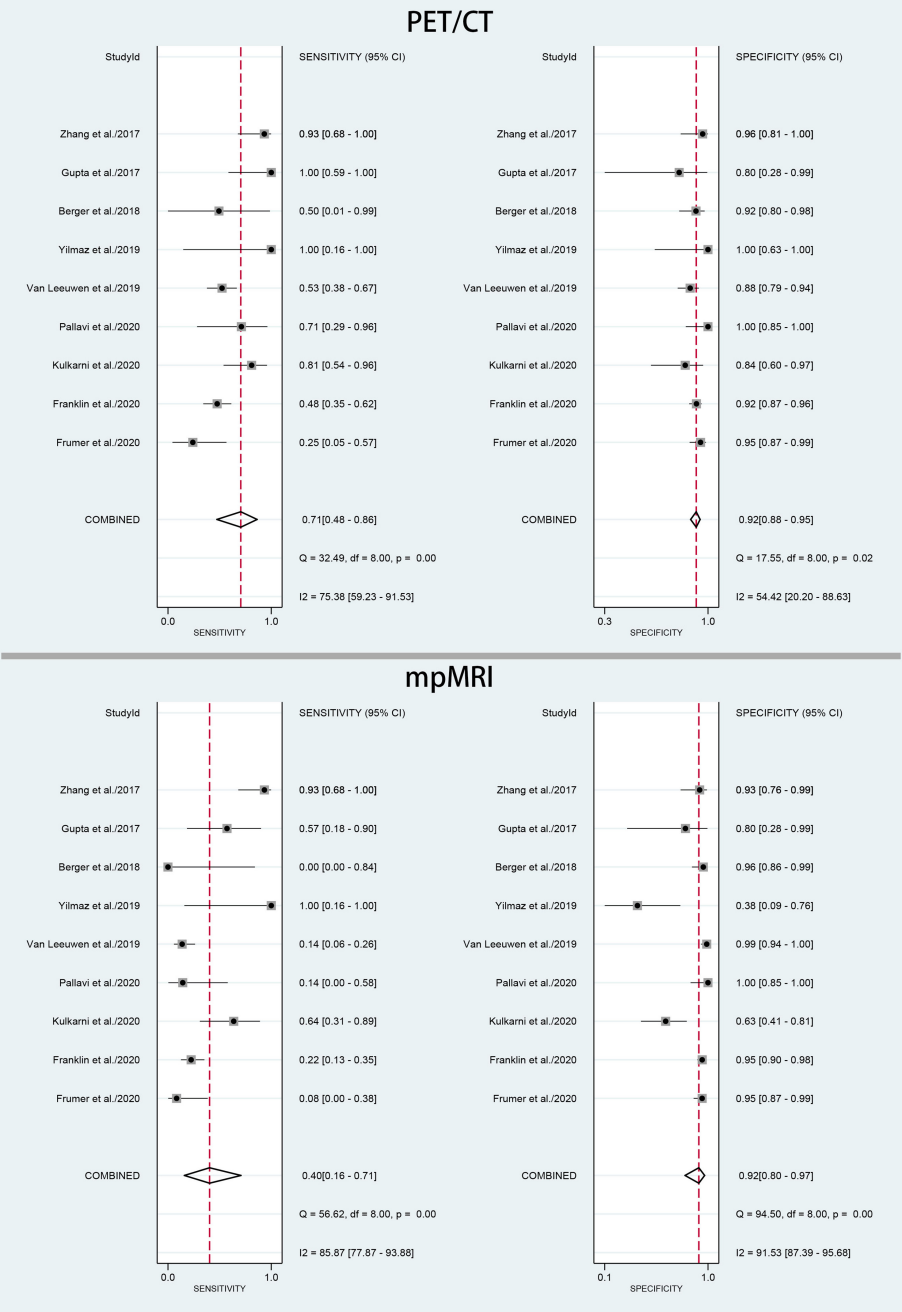
Forest plot of pooled sensitivity and specificity of ^68^Ga-PSMA-PET/CT and mpMRI for the detection of pelvic lymph node metastases prior to radical prostatectomy in PCa patients.

**Figure 4:**
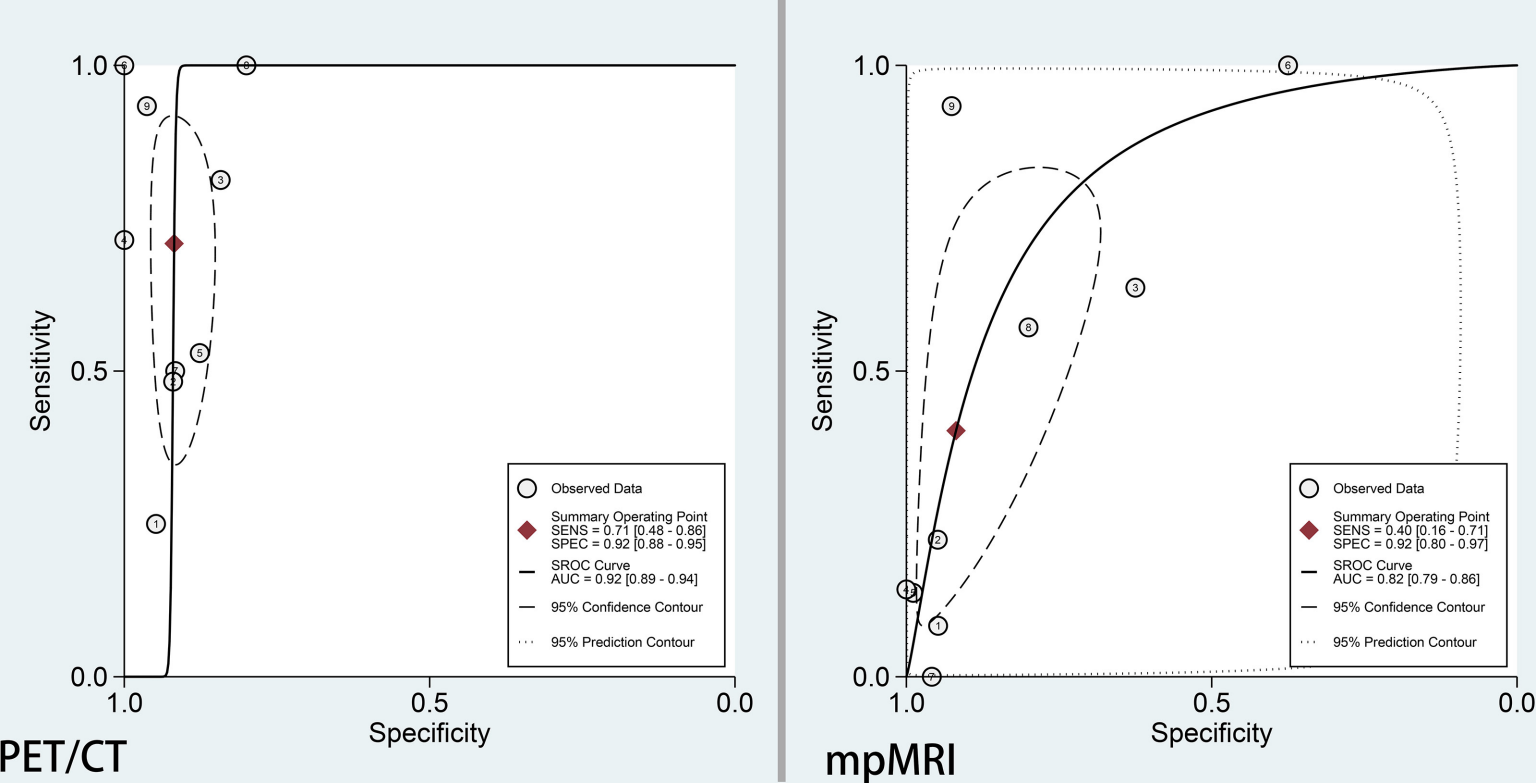
SROC curve of ^68^Ga-PSMA-PET/CT and mpMRI for the detection of pelvic lymph node metastases prior to radical prostatectomy in PCa patients.

The pooled sensitivity and specificity for ^68^Ga-PSMA-11 PET/CT were 0.71 (95% CI: 0.48–0.86) with moderate heterogeneity (75%) and 0.92 (95% CI: 0.88–0.95) with moderate heterogeneity (54%), respectively (**Figure 3**). **Figure 4** shows the SROC curve and the AUC for ^68^Ga-PSMA-11 PET/CT was 0.92 (95% CI: 0.89–0.94).

Meta-regression analysis was performed to explore the sources of heterogeneity, and we identified that prevalence of PLNMs (p = 0.01 for specificity), PSA level (p < 0.001 for sensitivity and p < 0.001 for specificity), risk classification (p < 0.001 for sensitivity), and reference standard (p < 0.001 for specificity) were possible causes of heterogeneity for ^68^Ga-PSMA-11 PET/CT. No publication bias was found (p = 0.15).

### Diagnostic Performance of mpMRI for PLNMs

The pooled sensitivity and specificity for mpMRI were 0.40 (95% CI: 0.16–0.71) with high heterogeneity (86%) and 0.92 (95% CI: 0.80–0.97) with high heterogeneity (92%), respectively (**Figure 3**). **Figure 4** shows the SROC curve and the AUC for mpMRI was 0.82 (95% CI: 0.79–0.86).

Meta-regression analysis revealed that number of patients (p < 0.001 for specificity) and PSA level (p < 0.001 for sensitivity) were possible causes of heterogeneity. No publication bias was found (p = 0.87).

## Discussion

**Figure 5:**
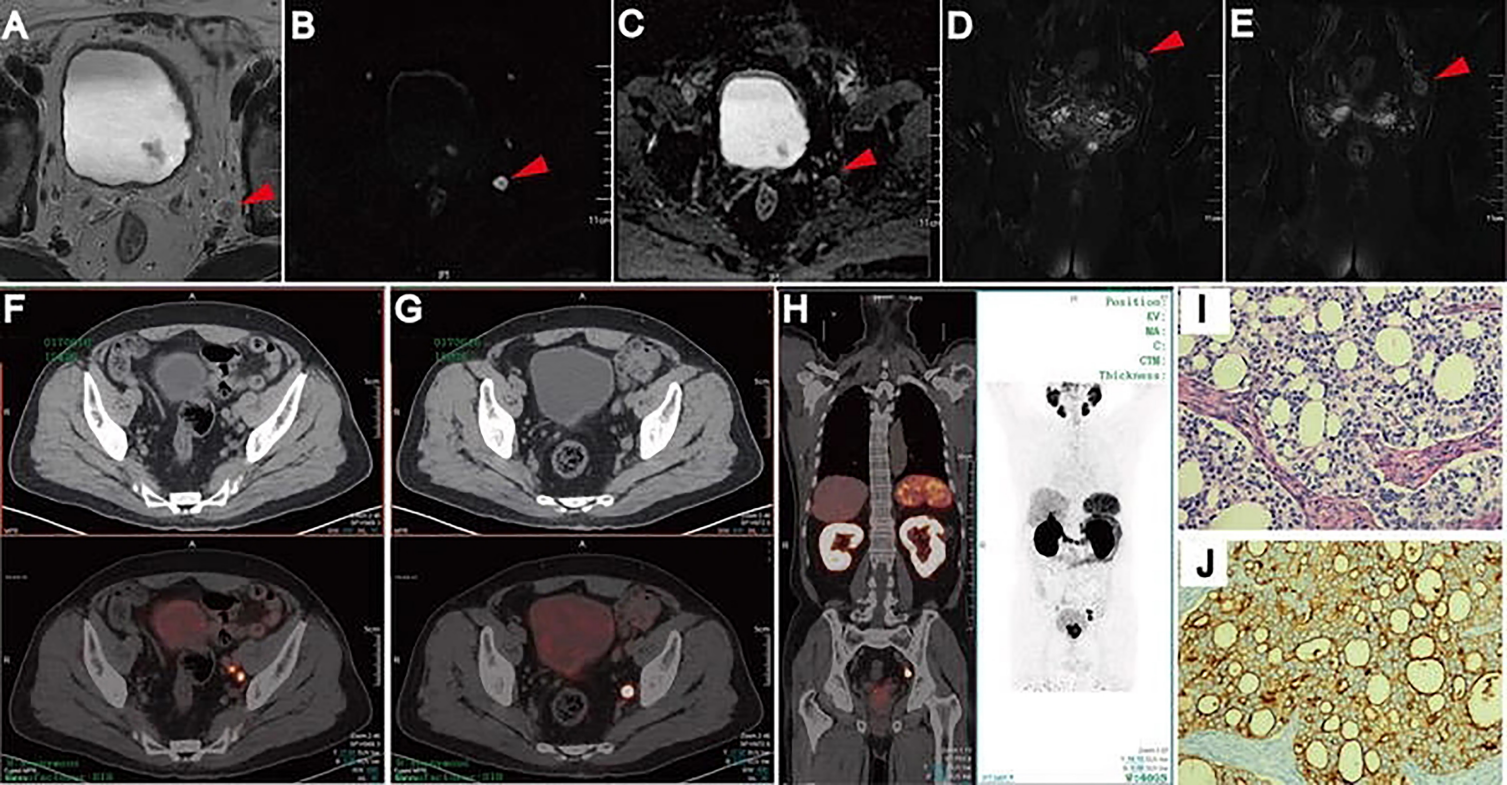
Lymph node metastases on pelvic mpMRI and 68Ga-PSMA PET/CT. Axial T2WI **(A)**, DWI **(B)**, ADC **(C)**, and coronal Fat suppression T2WI **(D, E)**. Fused 68Ga-PSMA PET/CT **(F–H)** images were taken from left internal iliac and obturator fossa regions with histopathologically proven disease (HE staining, **(I)** PSMA IHC staining, **(J)**. Reproduced with permission from **Figure 2** of Zhang et al. (19).

The present meta-analysis pooled patient-based data from nine studies which compared ^68^Ga-PSMA-11 PET/CT and mpMRI in the same population. It was found that the former had higher sensitivity (0.71 vs. 0.40), similar specificity (0.92 vs. 0.92), and higher AUC (0.92 vs. 0.82) as compared with the latter. The resulting relativeness was in agreement with those (sensitivity, 0.65 vs. 0.41; specificity, 0.94 vs. 0.92; AUC, 0.92 vs. 0.83) from a previous meta-analysis, in which indirect comparisons (not in the same population) were made by including 13 studies (29). The higher trend of sensitivity and diagnostic accuracy of ^68^Ga-PSMA-11 PET/CT over mpMRI for pelvic lymph node staging prior to radical prostatectomy in patients with intermediate to high-risk PCa were thus confirmed based on the most recent evidence. To better illustrate the imaging features of mpMRI and 68Ga-PSMA PET/CT in characterizing lymph node metastases, an example of one patient who had underwent both imaging modalities was shown in **Figure 5**.

Different interpreting strategies for small PLNMs between the two imaging modalities across the included studies might help to explain the better performance of ^68^Ga-PSMA-11 PET/CT. While most of the mpMRI interpretations used the short-axis diameter of more than 10 or 8 mm as a determining factor for malignancy, all PET/CT interpretations decided PLNMs solely based on PSMA uptake, irrespective of the small size of lymph nodes. Thus, some small PLNMs without significant anatomical characteristics might be only detected by PET/CT. In a study of 240 patients, Franklin et al. found that the median diameter of avid lymph nodes on ^68^Ga-PSMA PET/CT were 7.0 mm (range, 0.5–40 mm), in comparison to 11.7 mm (range, 2.2–20 mm) for mpMRI. The per-patient sensitivity of PET/CT and mpMRI in this study was 48.3% and 22.4%, respectively (32).

Nevertheless, ^68^Ga-PSMA-11 PET/CT still missed as many as 29% of the PLNMs identified by PLND according to the result of our meta-analysis. In a study of 140 patients, Van Leeuwen et al. reported that no lymph nodes detected < 2 mm and only 27% of the lymph node metastases 2 and 4 mm were detected by preoperative ^68^Ga-PSMA-PET/CT (24). In a larger study of 208 patients, Yaxley et al. found that 85.4% of histologically positive LNs ≤ 5 mm in maximal diameter were missed by preoperative ^68^Ga-PSMA PET/CT (34). It seems that the resolution of ^68^Ga-PSMA PET/CT is still not sufficient to detect many microscopic diseases seen at histopathology, particularly those with a diameter <5 mm. However, since it has been reported that the presence of microscopic diseases is associated with late disease recurrence, similar to PLNMs with large diameter, the clinical impact of these radiographically undetected microscopic diseases could be significant (35, 36). Therefore, despite its known limitations and complications, PLND remains necessary in that it could reveal microscopic diseases that might lead to early initiation of salvage radiotherapy and androgen deprivation therapy, which would eventually result in improved long-term local pelvic control and improved biochemical-free progression (2, 37).

**Figure 6:**
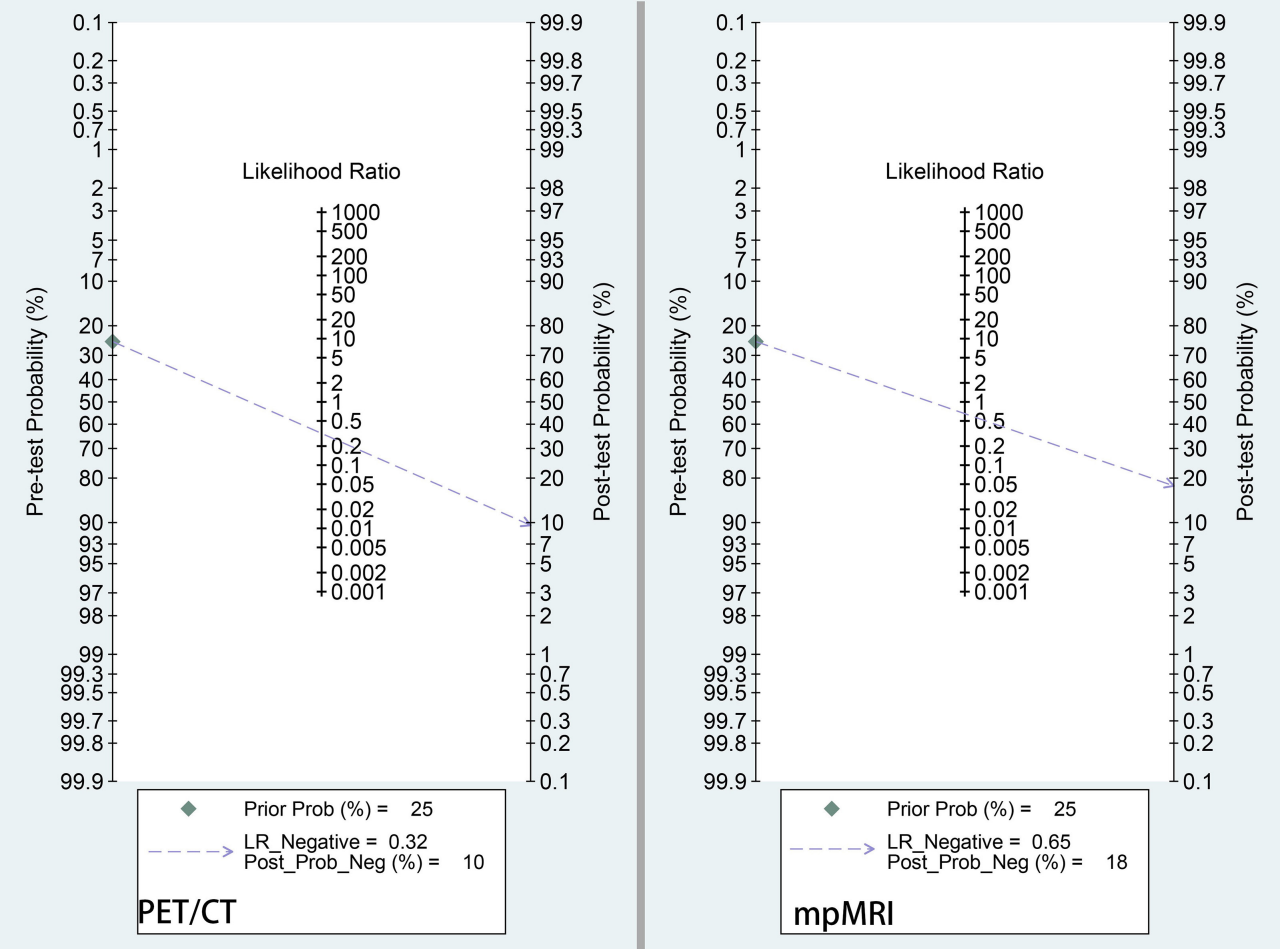
Fagan nomogram of pretest probability and negative posttest probability for ^68^Ga-PSMA-PET/CT and mpMRI. The pretest probability was set at 25%.

On the other hand, according to the current EAU or NCCN guidelines, if the risk of a PLNM is >5% or >2%, respectively, PLND is recommended at the time of radical prostatectomy (38, 39). Based on the results of this meta-analysis, Fagan’s nomogram indicated that when the pretest probability (prevalence of PLNMs) was assumed to be 25%, which is the medium value of our included studies, the negative posttest probability (the probability of being malignancy when the test is negative) decreased to 10% for ^68^Ga-PSMA-11 PET/CT and 22% for mpMRI (**Figure 6**). Thus, negative test results from both imaging modalities leaves a residual malignancy risk of above 5%. In this regard, PLND still needs to be recommended if ^68^Ga-PSMA PET/CT or mpMRI did not identify any suspicious lymph nodes.

In recent years, researchers have begun to incorporate ^68^Ga-PSMA PET/CT and mpMRI parameters into comprehensive preoperative algorithms to evaluate the risk of PLNMs. Franklin et al. found that the combination of a negative ^68^Ga-PSMA PET/CT, ISUP biopsy grade <4 and PIRADS <4 prostate mpMRI, or an ISUP grade 5 with PIRADS <3 on mpMRI was associated with a <5% risk of PLNMs (32). Ferraro et al. devised a model based on visual lymph node status on ^68^Ga-PSMA PET/CT, total PSMA uptake of the primary tumor, PSA, and Gleason score, which showed a tendency to improve patient selection for PLND overprediction models using clinical risk factors (40). It is hoped that future nomograms incorporating not only clinical risk factors but also data from modern imaging modalities will help to more appropriately select candidates for PLND. Moreover, hybrid PET/MRI modality may offer incremental value for preoperative detection of PLNMs. In a 2018 study, Thalgott et al. demonstrated that ^68^Ga-PSMA-11 PET/MRI even had a specificity of 100% in this setting (41).

Major limitations of our study include small sample size and heterogeneous study and patient characteristics and technical aspects of the included studies. We tried our best to perform subgroup analyses and found that number of patients, prevalence of PLNMs, PSA level, reference standard, and risk classification might be the sources of heterogeneity for the two imaging modalities. Besides, we only analyzed patient-based data in the present meta-analysis, because in clinical practice, it is difficult to precisely associate either PET or MRI findings with the histological results in a node-to-node manner and patients with one positive PLNM could provide enough prognostic information to alter patient management (34).

In conclusion, this meta-analysis of head-to-head comparison studies confirms that there is a trend toward a higher sensitivity and diagnostic accuracy of ^68^Ga-PSMA-11 PET/CT compared to mpMRI for the detection of PLNMs in PCa patients. Nevertheless, according to current guidelines, PLND still needs to be recommended in case of negative results from ^68^Ga-PSMA-11 PET/CT due to significant risk of malignancy. Hybrid PET/MRI modality exploiting both the superb molecular information from ^68^Ga-PSMA-11 PET and the high local contrast of MRI may represent a future direction.

## Data Availability Statement

The original contributions presented in the study are included in the article/supplementary material. Further inquiries can be directed to the corresponding authors.

## Author Contributions

JB and HZ conceived and designed the study, which were proofed by JB. XW, QW, and FT collected and analyzed the data. XW and QW wrote the manuscript. All authors contributed to the article and approved the submitted version.

## Conflict of Interest

The authors declare that the research was conducted in the absence of any commercial or financial relationships that could be construed as a potential conflict of interest.

## Publisher’s Note

All claims expressed in this article are solely those of the authors and do not necessarily represent those of their affiliated organizations, or those of the publisher, the editors and the reviewers. Any product that may be evaluated in this article, or claim that may be made by its manufacturer, is not guaranteed or endorsed by the publisher.
